# Safety and feasibility of countering neurological impairment by intravenous administration of autologous cord blood in cerebral palsy

**DOI:** 10.1186/1479-5876-10-58

**Published:** 2012-03-23

**Authors:** Young-Ho Lee, Kyung Vin Choi, Jin Hwa Moon, Hyun-Joo Jun, Hye-Ryeong Kang, Se-In Oh, Hyung Sun Kim, Jang Soo Um, Mi Jung Kim, Yun Young Choi, Young-Jun Lee, Hee-Jin Kim, Jong-Hwa Lee, Su Min Son, Soo-Jin Choi, Wonil Oh, Yoon-Sun Yang

**Affiliations:** 1 Department of Pediatrics & Cord Blood Clinic, Hanyang University Medical Center, Seoul, Korea; 2 Department of Rehabilitation Medicine, Hanyang University Medical Center, Seoul, Korea; 3 Department of Nuclear Medicine, Hanyang University Medical Center, Seoul, Korea; 4 Department of Radiology, Hanyang University Medical Center, Seoul, Korea; 5 Cell Therapy Center for Intractable Neurological Disorders, Hanyang University Medical Center, Seoul, Korea; 6 Department of Pediatrics, Wonkwang University Medical Center, Sanbon, Korea; 7 Department of Physical Medicine & Rehabilitation, Yeungnam University Medical Center, Daegu, Korea; 8 Medipost Biomedical Research Institute, Seoul, Korea

**Keywords:** Cerebral palsy, Cord blood, Mononuclear cells, Cell therapy

## Abstract

**Backgrounds:**

We conducted a pilot study of the infusion of intravenous autologous cord blood (CB) in children with cerebral palsy (CP) to assess the safety and feasibility of the procedure as well as its potential efficacy in countering neurological impairment.

**Methods:**

Patients diagnosed with CP were enrolled in this study if their parents had elected to bank their CB at birth. Cryopreserved CB units were thawed and infused intravenously over 10~20 minutes. We assessed potential efficacy over 6 months by brain magnetic resonance imaging (MRI)-diffusion tensor imaging (DTI), brain perfusion single-photon emission computed tomography (SPECT), and various evaluation tools for motor and cognitive functions.

**Results:**

Twenty patients received autologous CB infusion and were evaluated. The types of CP were as follows: 11 quadriplegics, 6 hemiplegics, and 3 diplegics. Infusion was generally well-tolerated, although 5 patients experienced temporary nausea, hemoglobinuria, or urticaria during intravenous infusion. Diverse neurological domains improved in 5 patients (25%) as assessed with developmental evaluation tools as well as by fractional anisotropy values in brain MRI-DTI. The neurologic improvement occurred significantly in patients with diplegia or hemiplegia rather than quadriplegia.

**Conclusions:**

Autologous CB infusion is safe and feasible, and has yielded potential benefits in children with CP.

## Backgrounds

Cord blood (CB) was introduced for the first time in humans to reconstitute the hematopoietic system in patient with Fanconi anemia [1]. Since the first cord blood transplantation (CBT), more than 20,000 CBTs have been reported worldwide and more than 400,000 CB units have been stored in more than 100 CB banks [2]. The clinical use of CB has expanded into various areas such as inherited metabolic disorders. CBT for Hurler syndrome resulted in either stabilization or improvement of neurocognitive function, and maintenance of new skills [3]. CBT for infantile Krabbe disease was highly effective if patients received transplants early in the course of the disease [4]. In such patients, CBT can prevent demyelination in the central and, often, the peripheral nervous system, extending life and improving overall quality of life. Kurtzberg, et al found that donor cells could enter the brain and induce remyelination and improvement in neurologic function in demyelinating diseases.

Cerebral palsy (CP) describes a group of permanent disorders of movement and posture limiting activity, due to non-progressive disturbances that occurred in the developing fetal or infant brain [5]. The ultimate goal of any therapy program for CP is to help children achieve their maximum potential in the motor, cognitive, and social realms. Despite a wide range of medical and surgical interventions in children with CP, and at risk of CP, there is no cure and significant variability in outcome, in part due to the heterogeneous nature of the underlying brain pathology.

Recently, the clinical application of CB in regenerative medicine has expanded using mesenchymal stem cells (MSC) and mononuclear cells (MNC). Since CB contains hematopoietic stem cells as well as a mixture of multipotent stem cells, such as unrestricted somatic stem cells, mesenchymal stem cells, and endothelial colony-forming cells, CB has the ability to regenerate numerous tissue types and improve their function. The evidence that CB cells express neurotrophic factors and produce cytokines that may be partially responsible for the functional brain repair, has prompted investigation of the therapeutic use of CB in various neurologic diseases [6,7]. MSC and MNC have been administered intrathecally and intravenously in experimental and clinical trials for neurologic disorders [8-11]. However, there is no definitive evidence concerning the optimum route for cell therapy. Intravenous infusion of autologous CB MNC in children with CP represents a novel and safe challenge that may involve a quite different mechanism of action from previous treatment methods.

We have conducted a single arm pilot study of intravenous autologous CB MNC infusion in children with CP to assess the safety and feasibility of the procedure as well as any effect in improving neurological function.

## Methods

### Patients

The study was approved by our institutional research ethics committee, and written informed parental consent was obtained for all patients. Twenty young patients aged 2~10 years and diagnosed with CP due to various causes were enrolled since their parents had elected to bank their CB privately (Medipost Biomedical Research Institutes, Seoul, Korea) at birth; patients with epilepsy were excluded. The participants were not provided with any additional medication or rehabilitation programs and for ethical reasons there was no control group. The diagnoses of CP were based on history taking, routine laboratory tests, genetic and metabolic studies, chromosome analysis, and neurological examination. To confirm the diagnoses, 2 clinicians, a pediatrician and a physiatrist, examined the patients. The major signs that collectively led to the diagnosis of CP were delayed motor milestones, abnormal neurologic examination, persistence of primitive reflexes, and abnormal postural reactions.

### Study protocol

Cryopreserved CB was delivered from the CB bank and infused intravenously over 10~20 minutes after thawing at the bedside, followed by administration of hydration fluid for 4~6 hours. To assess the short-term safety of CB infusion, we monitored vital signs and checked for symptoms such as fever, chills, nausea, and vomiting that could be caused by toxicity of the cryoprotectant and/or by lysis of red blood cells upon thawing the CB. The whole volume of thawed CB was infused and its cellular content then analyzed to enable evaluation of the impact of cell dose on clinical improvement. To assess the long-term safety of CB infusion and to measure neurodevelopmental changes, all the CP patients were followed up in the departments of pediatrics and rehabilitation medicine on the 4th, 8th, 12th, 24th weeks after infusion of CB.

The neurodevelopmental tests were carried out each time along with measurements of vital signs, weight and height, in addition to a complete blood cell count, urinalysis, and blood chemistry. Comparative analyses of brain perfusion single-photon emission computed tomography (SPECT), and magnetic resonance imaging (MRI) with diffusion tensor imaging (DTI), were performed at 24 weeks after infusion of CB.

### Neurodevelopmental examinations

Before and after the infusion of CB, all the CP patients were assessed by comprehensive neurodevelopmental examinations including the Denver development screening test II (DDST- II) as a gross developmental screening, the pediatric evaluation of disability inventory (PEDI) as a detailed developmental assessment, the gross motor function classification system (GMFCS) for gross motor function staging, the gross motor function measure-88 (GMFM) as a detailed motor function test, the manual ability classification system (MACS) for fine motor staging, and the quality of upper extremity skill test (QUEST) for fine motor function testing. The results for each examination tool were evaluated by well-trained physical and occupational therapists, and therapeutic responses were comprehensively assessed by rehabilitation specialists.

### Imaging studies

Brain perfusion SPECT, and conventional brain MRI-DTI were performed to evaluate functional changes of the brain. Fractional anisotropy (FA) values were obtained from the DTI data for 26 regions of interest (ROIs). All ROIs were set by the two pediatric neurologists, and all measurements were performed twice, and mean values used for analysis. We compared pre- and post-treatment FA values in the same patient. We also evaluated the correlation of FA value changes with neurodevelopmental changes. Brain perfusion SPECT using Tc-99 m ethyl cysteinate dimer was performed as a baseline study and as a follow-up study 24 weeks after infusion of CB. Brain perfusion SPECT images were evaluated visually by a nuclear medicine physician, and by semiquantitative analysis using an asymmetry index calculated as the relative uptake ratios in ROIs drawn in the frontal, temporal, parietal, and occipital cortices, basal ganglia and thalamus of both hemispheres.

### Statistical Analysis

In the analysis of FA values, a weighted kappa analysis was performed to measure inter-rater agreement. The Wilcoxon signed-rank test was used to compare differences of FA values pre- and post-treatment. In the comparative analysis of global outcome in the clinical characteristics and infused total nucleated cell (TNC) counts at 24 weeks after infusion, the variables were compared using Fisher's exact test and Wilcoxon rank-sum test. *P *values of ≤ 0.05 were considered significant. All statistical analyses were performed with SPSS (version 15.0, SPSS Inc, Chicago, IL, USA) statistical software.

## Results

Twenty patients (8 male, 12 female) received autologous CB infusion and were evaluated. Mean age and body weight at infusion were 55 (23~91) months and 13.9 (7.2~21.4)kg, respectively. Possible causes of the CP were: 8 hypoxic ischemic encephalopathies, 1 streptococcal meningitis, 1 middle cerebral artery infarction, 1 polymicrogyria, and 9 unknown. Types of CP were: 11 quadriplegia, 6 hemiplegia, 3 diplegia.

TNC of 5.5 ± 3.8 (0.6~15.65) × 10^7^/kg were infused. The viability of the cells before freezing was 95.4 ± 3.5%; it was not measured after thawing. Infusion was generally well-tolerated, even without premedication, although 3 patients experienced temporary nausea and hemoglobinuria, and another 2 patients experienced hemoglobinuria and urticaria, respectively, but these were easily controlled with peniramine or intravenous hydration. Although 14 of the 20 patients had increased scores in at least one of the 4 neurodevelopmental tests after CB infusion, only 5 patients (4 hemiplegia, 1 diplegia) showed more improvement in the neurodevelopmental tests than would normally be expected over a 6 month period. The neurodevelopmental improvements occurred significantly in patients with hemiplegia or diplegia rather than with quadriplegia (*p *= 0.008). Other variables, such as sex, age, body weight, infused TNC counts as well as possible causes of CP did not show any significant differences in global outcomes. Overall therapeutic responses according to clinical characteristics and infused TNC counts of CP patients are shown in Table 1.

**Table 1:** Therapeutic responses to autologous cord blood infusion according to clinical characteristics and infused TNC counts

UPN	Sex	Age (Mo)	BW (Kg)	TNC (10^7/kg)	Dx			Tx Response				
					
					Type	GA	Possible Causes	DDST II	PEDI	GMFCS	MACS	Overall
1	M	80	13	5.38	Quad	Full	Unknown	N	Y	N	N	N
2	F	24	10	6.72	Quad	Full	HIE, PVL	N	N	Y	N	N
3	F	91	14	10.92	Quad	Full	Unknown	N	Y	N	N	N
4	F	91	19.5	2.87	Quad	Full	HIE, MAS	N	N	N	N	N
5	F	82	18.1	4.36	Di	Full	Unknown	N	Y	N	N	N
6	F	28	7.2	9.58	Quad	Full	Unknown	N	N	N	N	N
7	M	31	11.2	5.72	Quad	Full	Strep meningitis	N	N	N	N	N
8	F	71	21.4	2.89	Hemi	Full	Polymicrogyria	Y	Y	N	N	Y
9	M	43	16.4	0.6	Hemi	Preterm	HIE, PVL	Y	Y	N	Y	Y
10	F	75	9.4	10.63	Quad	Full	HIE, MAS	N	N	N	N	N
11	F	53	15.3	5.88	Hemi	Full	Unknown	N	Y	N	N	Y
12	F	40	12.7	2.44	Hemi	Preterm	HIE, PVL	N	Y	N	Y	Y
13	M	29	10.8	6.85	Di	Preterm	HIE, PVL	Y	Y	N	N	Y
14	M	67	15	5.26	Quad	Full	Unknown	N	N	N	N	N
15	M	78	20	5.14	Hemi	Full	HIE, ICH, Infarction	Y	Y	N	N	N
16	F	71	17.9	2.86	Di	Full	Unknown	N	Y	N	N	N
17	F	58	11.6	15.65	Quad	Preterm	HIE, PVL	N	Y	N	N	N
18	M	29	11.3	0.71	Quad	Full	Unknown	N	Y	N	N	N
19	F	30	11.5	3.29	Hemi	Full	MCA infarction	N	N	N	N	N
20	M	23	10.9	2.25	Quad	Full	Unknown	N	Y	N	N	N

UPN; unique patient number, BW; body weight, TNC; total nucleated cells, Dx; diagnosis, Tx; treatment, GA; gestational age, DDST-II; Denver developmental screening test II, PEDI; pediatric evaluation of disability inventory, GMFCS; Gross motor function classification system, MACS; Manual ability classification system, Quad; quadriplegic, Di; diplegic, Hemi; hemiplegic, Full; full-term, HIE; hypoxic ischemic encephalopathy, PVL; periventricular leukomalacia, MAS; meconium aspiration pneumonia, Strep; streptococcal, ICH; intracranial hemorrhage, MCA; middle cerebral artery, N; no, Y; yes

In the analysis of FA values in MRI-DTI, significant differences between pre-treatment and post-treatment values were found in only 3 ROIs (right temporal, corpus callosum, and right periventricular white matter) out of 26. In the 5 patients who showed comprehensive improvements in the neurodevelopmental tests, we examined these areas to analyze the clinical significance of the FA value changes. The differences of white matter integrity (FA values) in ROIs of 5 patients who showed clinical improvement after autologous CB infusion are presented in Table 2.

**Table 2:** Differences of white matter integrity (FA values) of the 5 patients who showed neurodevelopmental improvement

ROIs		UPN 8		UPN 9		UPN 11		UPN 12		UPN 13	
		Pre	Post	Pre	Post	Pre	Post	Pre	Post	Pre	Post
Temporal	TR *	0.431	0.451	0.438	0.421	0.425	0.445	0.463	0.464	0.416	0.430
	TL	0.483	0.457	0.448	0.456	0.319	0.346	0.460	0.444	0.423	0.460
Orbito-frontal	FR	0.411	0.465	0.470	0.378	0.359	0.386	0.421	0.438	0.466	0.728
	FL	0.469	0.490	0.450	0.383	0.319	0.336	0.423	0.438	0.462	0.450
Inferior frontal	IFR	0.453	0.446	0.444	0.447	0.397	0.370	0.404	0.437	0.443	0.474
	IFL	0.506	0.515	0.494	0.383	0.320	0.334	0.457	0.464	0.452	0.454
Internal capsule	ICR	0.489	0.499	0.498	0.570	0.501	0.534	0.476	0.498	0.467	0.465
	ICL	0.527	0.520	0.493	0.504	0.336	0.328	0.496	0.496	0.478	0.454
	CC *	0.534	0.538	0.493	0.487	0.421	0.427	0.443	0.509	0.481	0.447
	S	0.502	0.536	0.472	0.495	0.421	0.421	0.512	0.549	0.471	0.465
Periventricular white matter	AWMR	0.374	0.366	0.363	0.300	0.363	0.369	0.363	0.307	0.373	0.332
	AWML	0.423	0.385	0.375	0.315	0.264	0.228	0.349	0.371	0.332	0.378
	PWMR*	0.479	0.479	0.400	0.425	0.426	0.481	0.407	0.415	0.398	0.408
	PWML	0.488	0.484	0.476	0.442	0.314	0.392	0.459	0.444	0.414	0.410
Occipital	OR	0.443	0.457	0.523	0.449	0.441	0.446	0.450	0.430	0.414	0.434
	OL	0.483	0.510	0.488	0.395	0.284	0.271	0.536	0.509	0.441	0.391
Sup frontal subcortex	SFR	0.475	0.447	0.484	0.444	0.346	0.330	0.345	0.431	0.452	0.433
	SFL	0.455	0.460	0.443	0.360	0.308	0.334	0.399	0.401	0.452	0.440
Middle frontal paravent WM	MFPAR	0.336	0.319	0.363	0.379	0.389	0.396	0.378	0.381	0.389	0.414
	MFPAL	0.455	0.447	0.433	0.389	0.263	0.237	0.385	0.387	0.387	0.438
Parietal perivent WM	PPER	0.468	0.451	0.447	0.440	0.466	0.504	0.446	0.479	0.435	0.445
	PPEL	0.519	0.514	0.458	0.456	0.342	0.350	0.453	0.458	0.446	0.453
Post parietal perivent WM	PPPER	0.467	0.507	0.405	0.467	0.477	0.495	0.492	0.488	0.391	0.476
	PPPEL	0.465	0.466	0.464	0.427	0.284	0.284	0.452	0.525	0.408	0.372
Post parietal paravent WM	PPPAR	0.445	0.497	0.336	0.412	0.480	0.477	0.433	0.473	0.425	0.508
	PPPAL	0.441	0.443	0.381	0.359	0.273	0.288	0.448	0.422	0.374	0.425

UPN; unique patient number, TR; right temporal, TL; left temporal, FR; right orbito-frontal, FL; left orbito-frontal, IFR; right inferior frontal, IFL; left inferior frontal, ICR; right internal capsule, ICL; left internal capsule, CC; corpus callosum, S; splenium, AWMR; right anterior periventricular white matter, AWML; left anterior periventricular white matter, PWMR; right posterior periventricular white matter, PWML; left posterior periventricular white matter, OR; right occipital, OL; left occipital, SFR; right superior frontal subcortex, SFL; left superior frontal subcortex, MFPAR; right anterior middle frontal paraventricular white matter, MFPAL; left anterior middle frontal paraventricular white matter, PPER; right parietal periventricular white matter, PPEL; left parietal periventricular white matter, PPPER; right posterior parietal periventricular white matter, PPPEL; left posterior parietal periventricular white matter, PPPAR; right posterior parietal paraventricular white matter, PPPAL; left posterior parietal paraventricular white matter (* indicates the ROIs demonstrated significant differences between pre-treatment and post-treatment values out of 26 ROIs.)

In the brain perfusion SPECT analysis, 2 (UPN 8,9) of the patients who experienced clinical improvement displayed improved perfusion. Both had mild forms of hemiplegia, and reduced perfusion in the left thalamus in the baseline SPECT studies, which increased in the SPECT images at 24 weeks. Six patients (UPN 6,12,13,14,15,16) showed decreased perfusion in the unilateral thalamus in the baseline SPECT images, and this expanded to the contralateral thalamus in the subsequent SPECT images. The rest of the patients (n = 12) showed no significant changes in cerebral perfusion state in SPECT images.

The detailed changes in the 5 patients in whom we detected neurological improvement after CB infusion are set out below.

UPN 8 (F/71 months old)

**Figure 1:**
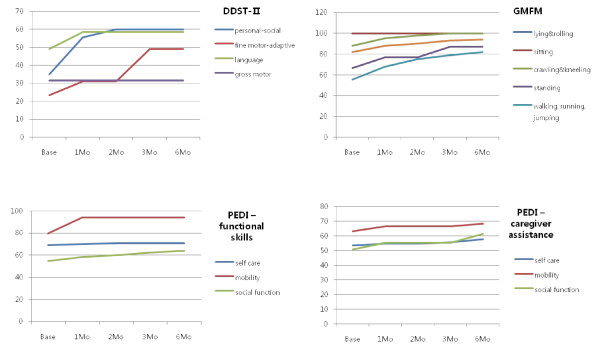
**Neurodevelopmental evaluation in UPN 8**. One month after cord blood infusion, personal-social, fine motor-adaptive, and language scores in DDST-II improved. In GMFM, most domains improved gradually, while in PEDI, social function improved slightly. DDST-II; Denver developmental screening test II, PEDI; pediatric evaluation of disability inventory, GMFM; Gross motor function measure-88.

**Figure 2:**
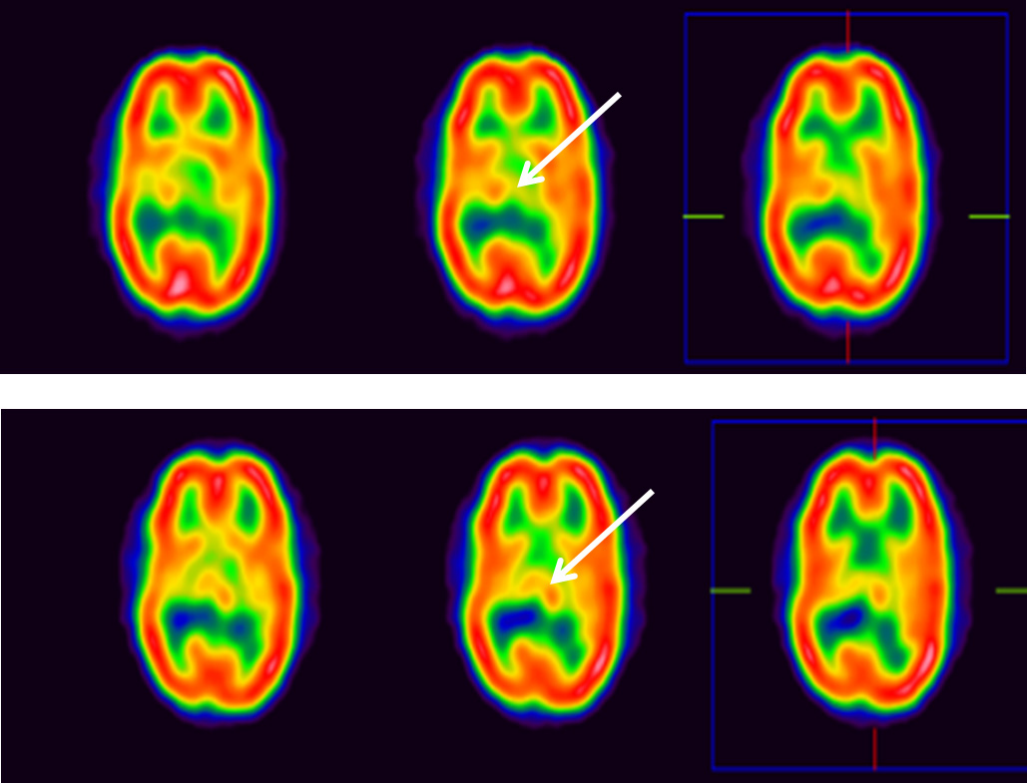
**Brain perfusion SPECT analysis**. The low perfusion in the left thalamus in the baseline study (white arrow, upper lane), has been corrected in subsequent images at 24 weeks (white arrow, lower lane) after infusion of cord blood.

This child had functional hemiplegia and polymicrogyria on conventional brain MRI. One month after CB infusion, she improved on the personal-social, fine motor-adaptive, and language scales of DDST-II. She became able to dress herself and brush her teeth on the personal-social scale of DDST-II. Her score on the fine motor adaptive scale increased markedly in three months. She could make piles of 6 to 8 blocks, and was able to draw straight lines and circles. In GMFM, her balance while standing was enhanced as was her ability to jump over sticks and stand on one foot. In terms of PEDI-functional skills, she showed increased mobility and a slight improvement in social function. She also had slightly increased self-care, mobility, and social function in terms of problem solving, self-awareness and time orientation in the PEDI-caregiver assistant scale (Figure 1). In the analysis of MRI-DTI, we noted increments of FA values in the right temporal area, among the three significant areas. She displayed decreased perfusion in the left thalamus and right basal ganglia in the baseline SPECT study, and improved perfusion in these areas in subsequent brain perfusion SPECT images (Figure 2).

UPN 9 (M/43 months old)

**Figure 3:**
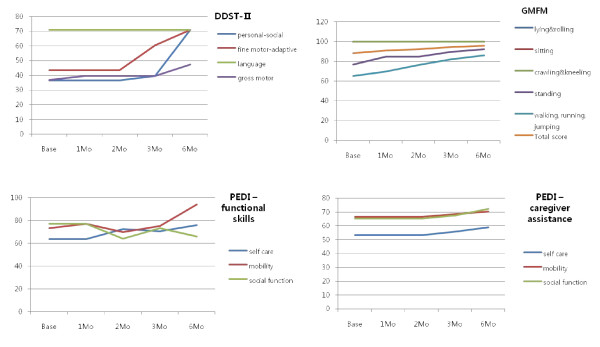
**Neurodevelopmental evaluation of UPN 9**. Fine motor-adaptive and personal-social function in DDST-II improved after 3 months. Walking, running, and total functional scores increased in GMFM. All factors in PEDI-caregiver assistance slightly improved. DDST-II; Denver developmental screening test II, PEDI; pediatric evaluation of disability inventory, GMFM; gross motor function measure-88.

This child was a preterm baby with hypoxic-ischemic encephalopathy, displaying weakness on his left side with periventricular leukomalacia. Fine motor-adaptive function such as circle drawing and personal-social functioning such as independent dressing improved 3 months after CB infusion, and this was followed at 6 months by improvements in brushing teeth and following the rules of games, as well as gross motor functions on the DDST-II scale. Walking, running, and standing abilities, along with total scores in the GMFM, increased consistently. In PEDI-functional skills, mobility, self-care, and social function remained stationary for 3 months after CB infusion. In PEDI-caregiver assistance, all factors increased slightly after 2 months (Figure 3). In the analysis of MRI-DTI, we noted an increment of FA value in the right periventricular white matter. Perfusion in left thalamus was reduced in the baseline SPECT images, but improved in subsequent SPECT images.

UPN 11 (F/53 months old)

**Figure 4:**
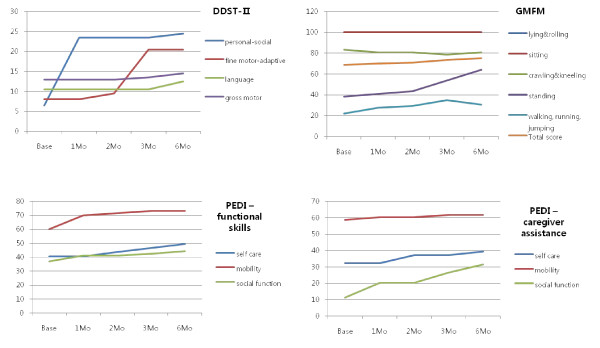
**Neurodevelopmental evaluation of UPN 11**. In DDST-II, the personal-social score increased dramatically by 1 month after infusion and fine motor-adaptive function also increased by 3 months after infusion. In PEDI, self-care and social function gradually increased. DDST-II; Denver developmental screening test II, PEDI; pediatric evaluation of disability inventory, GMFM; gross motor function measure-88.

This little girl was hemiplegic with no defined cause. Scores on the personal-social scale in DDST-II, in skills such as brushing teeth with assistance, undressing, using fork and spoon, and tidying toys, increased dramatically 1 month after CB infusion, and fine motor-adaptive activities such as tapping an object with either hand, picking a plum from a bottle and piling up pairs of blocks began to increase after 2 months. Standing ability in GMFM increased after the second month, and there was a gradual increase in functional skills and caregiver assistance in PEDI self-care activities such as using utensils, grooming, washing her face and bowel management, and in social functions including language comprehension and playing with toys (Figure 4). In the analysis of MRI-DTI, an increment of FA values in the right posterior periventricular white matter and right periventricular white matter was noted. Perfusion in the left posterior frontal and upper temporal cortexes, left basal ganglia, left thalamus and left cerebellum was reduced in the baseline SPECT images, and did not change significantly in subsequent SPECT images.

UPN 12 (F/40 months old)

**Figure 5:**
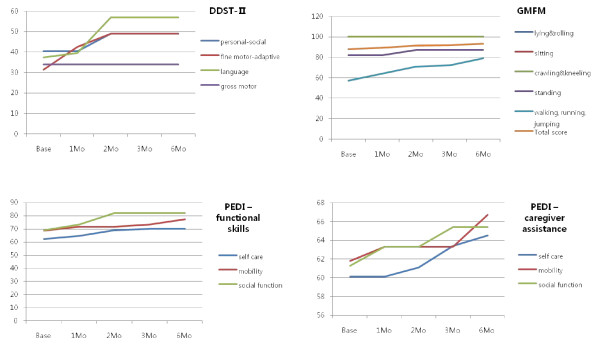
**Neurodevelopmental evaluation of UPN 12**. All functions except gross motor in DDST-II improved by 2 months after infusion. Walking and running steadily improved in GMFM. Self-care, mobility, and social functions increased gradually on PEDI-caregiver assistance scale. DDST-II; Denver developmental screening test II, PEDI; pediatric evaluation of disability inventory, GMFM; gross motor function measure-88.

This little girl was a preterm baby with hypoxic-ischemic encephalopathy, and displayed weakness on her left side with periventricular leukomalacia. All functions except gross motor function in DDST-II improved by 2 months after CB infusion. She could dress herself and follow the rules of games on the personal-social scale, and was also able to use building blocks, draw circles, drawing a person and distinguish two straight lines on the fine motor-adaptive scale. Language improved markedly after the first month. She became able to understand the identities of objects, prepositions and adjectives on the language scale. Walking and running gradually improved on GMFM, and all tests showed a gradual increase in PEDI-functional skills. Self-care, mobility, and social function increased dramatically on the PEDI-caregiver assistance scale (Figure 5). In the analysis of MRI-DTI, increments of FA values in the right temporal and corpus callosum were noted. There was decreased perfusion in the bilateral anterior medial frontal cortex, and a suspicion of decreased perfusion in the bilateral thalamus on the baseline SPECT images, and this was followed by a definite decrease of perfusion in the bilateral thalamus on subsequent SPECT images.

UPN 13 (M/29 months old)

**Figure 6:**
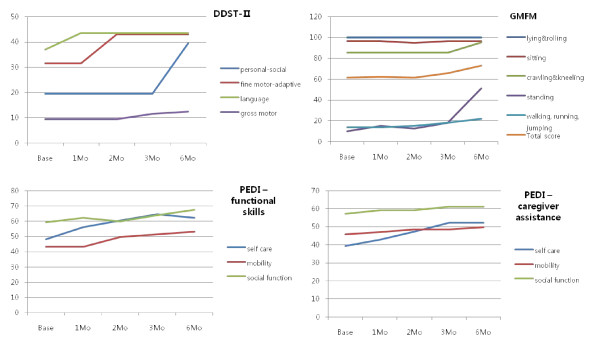
**Neurodevelopmental evaluation of UPN 13**. By 1 month after cord blood infusion, language had improved, and in DDST-II there were step-wise increments in fine motor-adaptive functions and personal-social skills in the following months. In GMFM, standing definitely improved after the third month. In PEDI, all scores increased gradually. DDST-II; Denver developmental screening test II, PEDI; pediatric evaluation of disability inventory, GMFM; Gross motor function measure-88.

This male child was diplegic and preterm with periventricular leukomalacia. One month after CB infusion language improved, and fine motor-adaptive functions followed after the second month. After the third month, personal-social skills including ability to undress, dress, brush teeth with help, put on a shirt and say the names of friends improved in DDST-II. In GMFM, standing improved dramatically, along with improvements in crawling and kneeling, as well as walking and running, after the third month. He was able to attempt to stand on alternate feet. In PEDI, all scales increased gradually. Improvements were achieved in using utensils, maintaining self-hygiene, bathing, bowel and bladder management, and problem-solving ability and time-orientation also improved (Figure 6). In the analysis of MRI-DTI, an increment of FA values in the right temporal area was noted. There was reduced perfusion in the right thalamus in the baseline SPECT image, and perfusion worsened in the bilateral thalamus in the subsequent SPECT images.

## Discussion

We observed that 25% of children with CP showed partial improvements in neurodevelopmental evaluation tests following intravenous, autologous CB MNC infusion. Interestingly, neurologic improvement was seen in patients with diplegia or hemiplegia rather than with quadriplegia, although this study is limited by the small number of subjects as well as the different types of CP and underlying causes. Brain plasticity is another limitation of this trial. However, we attempted to reduce any confounding effects of brain plasticity by including only patients between 2 and 10 years of age, to minimize the effect of brain plasticity during the first few years of life, and at the same time to maximize the kilogram-based cell dose of infused CB. We also demonstrated that age did not significantly affect global outcomes.

Abnormalities are demonstrable in many children with CP by conventional MRI, and DTI can be used to visualize brain white matter tracts, and has been shown to improve the detection of lesions in a range of neurologic disorders [12]. Trivedi, et al demonstrated a correlation between clinical grade and DTI measurements in motor and sensory pathways in children with CP [13]. Yoshida, et al demonstrated that ROI-based FA values in the corticospinal tract and posterior thalamic radiation were significantly lower in children with CP than in control children, and they suggested that the DTI parameters of corticospinal tract and posterior thalamic radiation are useful variables for evaluating clinical motor status and outcomes in children with CP [14]. Hoon, et al also showed a significant correlation between DTI scores in thalamocortical pathways and the quantitative clinical status of children with CP [15]. We also analyzed MRI-DTI data to obtain imaging evidence of neurologic improvements. Based on findings from the aforementioned studies, we chose 24 weeks after CB infusion as the time for this analysis. Because we did not use control MRI-DTI data from healthy volunteers, we attempted to identify ROIs in which there were significant differences of FA values between pre- and post-treatment. We found that FA values in only 3 of the total of 26 ROIs displayed significant differences between pre- and post-treatment in the 19 evaluable patients. We assessed the correlation between clinical changes and changes in FA values in these 3 ROIs, in the 5 patients who showed neurodevelopmental improvements. The improved language scale and self awareness in UPN 8 may have been related to the increased FA values in the temporal lobe, but this latter cannot fully explain the overall improvements in the fine motor and social areas. FA values in the right periventricular white matter area increased in UPN 9 and could underlie his improvement in GMFM and fine motor-adaptive function. In UPN 11, the MRI-DTI changes involving partial increases in the anisotropy of the temporal lobe and posterior thalamic radiation could be related to her improved language function and increased score in mobility and self care ability. FA values in the corpus callosum increased in UPN 12; the corpus callosum is a highly dense pathway of white matter connecting right and left hemispheres, and the increase of FA after treatment could indicate improvement in the connectivity of the corpus callosum that permitted more effective transfer of information between the two sides of the brain. In UPN 13, the increase in FA values in the right temporal area might explain the improved functioning of the language area, but does not account for the increased fine motor function, mobility and other overall improvements.

In the brain perfusion SPECT analysis, 2 of the patients who showed clinical improvement displayed improved perfusion in thalamic areas, while regions of decreased perfusion, mainly in thalamic areas, expanded in another 6 patients. However, small, deep-seated structures such as the thalamus are susceptible to artifacts in SPECT images caused by attenuation of the low energy gamma rays emitted by these structures. Decreased image activity can be caused by increased attenuation, so that subtle changes can be artifactual. Further studies using brain positron emission tomography images generated by higher energy gamma rays could provide more reliable data on small, deep-seated structures.

Most studies of tissue regeneration have been performed by injecting MSC into target organ, although a variety of stem cell sources and routes of administration have been used in both animal models and humans. A double-blind, randomized, controlled trial revealed that bone marrow-derived MSC may be more effective than bone marrow-derived MNC in increasing lower limb perfusion and promoting foot ulcer healing in diabetic patients [16]. However, intravenously injected MNC obtained from the bone marrow of rats also induced functional recovery and decreased neurodegeneration after sensorimotor cortical ischemia in rats [17]. Reich, et al also showed that heterogeneous MNC and even CD133-depleted fractions have the ability not only to reduce apoptosis in neuronal cells but also to induce the maintenance of neuronal phenotypes [18].

The potential therapeutic benefits of human CB cells for treating injuries, diseases, and neurodegeneration are becoming increasingly recognized. The mechanisms underlying functional recovery remain to be clarified. Although Chen, et al observed that intravenously administered CB cells entered the brain, survived, migrated, and improved functional recovery after stroke [9], few CB cells are present in ischemic region compared to the number that were infused [19,20]. The small number of neural cells derived from the CB cells appears unable to explain the significant improvement resulting from cell transplantation. Kurtzberg observed neurological improvements in children with inborn errors of metabolism following intravenous infusion of CB in allogeneic CBTs, and detected the transplanted donor cells in recipients' brains [4]. Based on these experiences, she examined the effect of autologous CB infusion on neurological function in children with CP and again observed functional improvements [21].

However, in contrast to conventional hematopoietic stem cell transplantation with CB, non-hematopoietic applications such as cardiovascular or neurological indications do not require permanent graft survival because the therapeutic activities of the CB are believed to be mediated in many cases by growth factor secretion [22,23]. Fan, et al found significantly higher levels of brain-derived neurotrophic factors (BDNF) and neurotrophin-4/5 in the culture supernatants of CB MNC than of peripheral blood MNC [6].

The neurologic improvement we observed in our study may be due to some migration of MNC into the brain and the consequent cellular effects as well as above-mentioned neurotrophic effects of the CB. Intravenously administered CB cells would not enter the brain in CP patients without some challenge such as chemo-radiotherapy which could induce permeability changes in the blood brain barrier (BBB). Most studies of intravenous infusion of MNC to restore neurologic function have been performed in acute stages of neurological impairment when the BBB is usually disrupted. However, since Newman, et al have demonstrated that interleukin (IL)-8, monocyte chemotactic protein (MCP)-1, and IL-1α are consistently expressed by CB MNC regardless of culture conditions [7], the cytokines in CB which have been cited as pro-inflammatory mediators may be able to affect endothelial cells in such a way as to cause alterations in tight junction structure, the BBB, and leukocyte migration [24]. Microglia, which differentiate from monocytes, are major players in neurogenesis [25]. In inflammatory conditions, there can be both intrinsic proliferation of parenchymal microglia and substantial recruitment of monocytes [26]. Although studies have shown that inflammation and microglial activation can be detrimental to adult neurogenesis, their effects have turned out to be more complex and there is recent experimental evidence that they can be beneficial under some circumstances and support various steps in adult neurogenesis. It is conceivable that the early detrimental effects of microglia after acute injury can in some situations be converted into a supportive role during the chronic phase [27]. Microglia could then release neurotrophic factors such as BDNF and glial cell line-derived neurotrophic factor (GDNF), remove synapses from damaged neurons and influence the synaptic connectivity of newly-formed neurons [28].

## Conclusion

Autologous intravenous CB MNC infusion seems to be practicable and safe and has yielded potential benefits in children with CP. Further clinical studies including randomized, cross-over, long-term follow-up trials, as well as basic research into the underlying mechanisms, are needed.

## Abbreviations

CB: cord blood; CP: cerebral palsy; MRI: magnetic resonance imaging; DTI: diffusion tensor imaging; SPECT: single-photon emission computed tomography; CBT: cord blood transplantation; MSC: mesenchymal stem cells; MNC: mononuclear cells; TNC: total nucleated cell; DDST-II: Denver development screening test II; PEDI: pediatric evaluation of disability inventory; GMFCS: gross motor function classification system; GMFM: gross motor function measure-88; MACS: manual ability classification system; QUEST: quality of upper extremity skill test; FA: fractional anisotrophy; ROI: regions of interest; IL: interleukin; MCP: monocyte chemotactic protein; BBB: blood brain barrier.

## Competing interests

The authors declare that they have no competing interests.

## Authors' contributions

YHL conceived and participated in the design of the study and drafted manuscript. KVC participated in its design and statistical analysis and also helped to draft the manuscript. JHM, JHL, YJL, HJK, YYC and SMS participated in the radiologic data analysis and interpretation. HSK, JSU, SIO and MJK participated in the evaluation and interpretation of neurologic functions. They also helped to draft manuscript. HJJ, HRK, SJC, WO and YSY participated in the coordination of patients and evaluations. All authors read and approved the final manuscript.
